# Aortic arch de-branching for suspected expanding perigraft haematoma after previous acute type-A dissection repair with AMDS stent: a technique for a potential future problem

**DOI:** 10.1186/s13019-024-02825-5

**Published:** 2024-06-21

**Authors:** Rickesh Karsan, Niamh Shearer, Ciara Doyle, Rachel Roberts, Alsir Ahmed

**Affiliations:** Department of Cardiothoracic Surgery, Royal Victoria Hospital, Belfast, BT12 6BA UK

**Keywords:** Acute stanford-a aortic dissection, Ascyrus medical dissection stent, Aortic arch

## Abstract

**Background:**

Acute Stanford type- A aortic dissections make up a large part of emergency cardiac surgery. They also carry a significant burden of morbidity. New techniques to aid aortic remodelling include the Ascyrus Medical Dissection Stent (AMDS): Its increasing use, looks to present a potential problem in cases where surgery involving the aortic arch may be required.

**Case report:**

We present the case of a 49-year-old male who underwent urgent redo-surgery for total arch replacement and de-branching following recent replacement of the ascending aorta for acute type-A dissection, where an AMDS stent was deployed. The patient underwent total arch replacement with a stented tri-furcate prosthesis and de-branching of arch vessels with the stent landed inside the previous AMDS, to good effect.

**Conclusion:**

This case highlights a possible approach to aortic arch surgery in patients who have previous had AMDS insertion.

## Introduction

Emergency surgery for Stanford-A aortic dissection (SAAD) carries a large burden of mortality and morbidity. There are different anatomical and technical variables to consider to each case, which ultimately dictate the extent of repair required, as such an individualised approach is often required. This ranges from root replacement all the way to total arch replacement. Closure of the false lumen should prevent the dissection from propagating further distally and result in remodelling of the aorta. However, it is noted that the intimal flap after conventional repair can create a new entry leading to false lumen pressurisation and collapse of the true lumen.

The introduction of the Ascyrus Medical Dissection Stent (AMDS), an uncovered self-expanding endovascular stent, placed in the true lumen at zone 0 has aimed to improve the standard of care in type A aortic dissection [1]. The device intends to limit and obliterate perfusion of the false lumen by sealing the distal anastomosis, thus promoting thrombosis and preventing distal anastomotic new entry (DANE). Mid-term results are strongly suggestive that AMDS facilitates positive remodelling of the aortic arch with safe and reproducible results shown in the Dissected Aorta Repair Through Stent Implantation trial (DARTS) [2, 3]. The use of the AMDS device can cause future problems. The device cannot be removed due to risk of damage to the native aorta. This poses significant technical difficulties when redo-surgery is required for the aortic arch.

We present a novel approach to aortic arch surgery in a patient who had a previously deployed AMDS who later required total arch replacement, to good effect.

## Case report

**Fig. 1:**
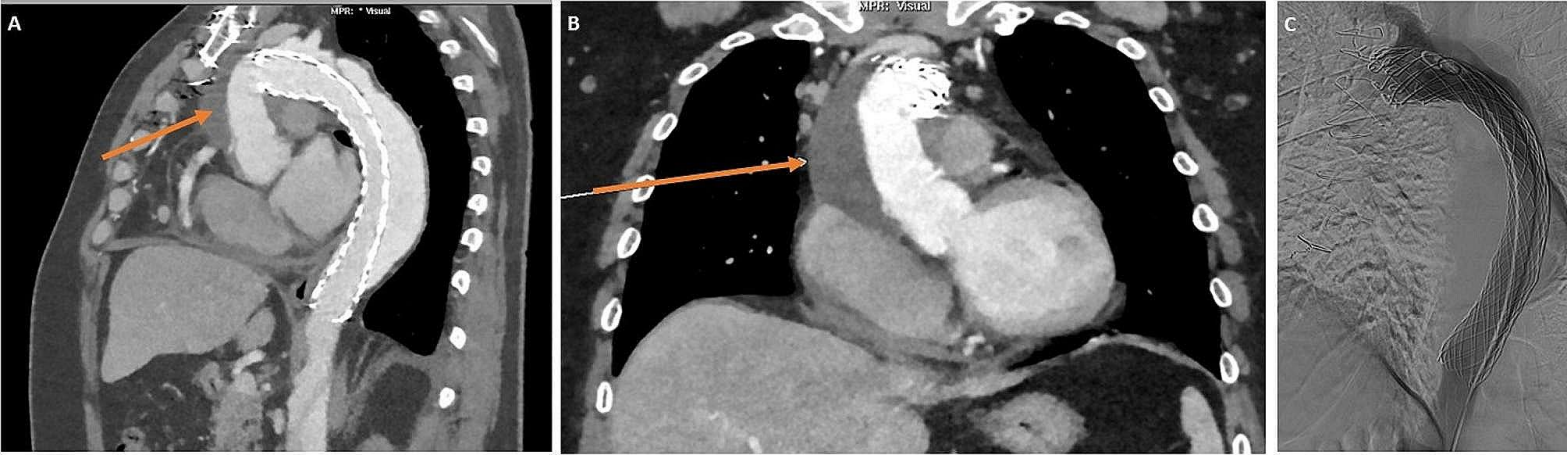
(**A-B**) CT aortogram showed an expanding perigraft haematoma (orange arrow). (**C**) Angiogram appearances suggestive of an ongoing leak at the proximal end of the stent, with opacification of the false lumen arising around the innominate artery origin and propagating through the descending thoracic aorta into the thoracoabdominal segment

A 49-year-old male, with a previous SAAD repair re-presented with new chest pain and was found to have a persistatnt false lumen. For his first presentation, he had a dissection involving his right coronary sinus of Valsalva, and was extending down to the iliac vessels. He underwent reconstitution of the aortic root, repair of the dissection flap, replacement of the ascending aorta with a size 28 mm GelWeave interposition tube graft and insertion of a 55 − 45 AMDS stent into the aortic arch and the proximal descending thoracic aorta. CT aortogram post-repair showed a persistent false lumen. Interval imaging illustrated a strong suspision of an expanding peri-graft haematoma from 4.5 cm to 6.5 cm over an 8 month period with noted incipid reduction in haemoglobin despite transfusion. Subsequent, diagnostic angiogram showed opacification of the false lumen, arising around the proximal end of the AMDS stent anastomosis, and was suggestive of ongoing leak and DANE with expanding false lumen (Fig. 1). The patient was discussed in a focused aortic multidisciplinary meeting and given the ongoing risk of an expanding perigraft haematoma and false lumen, an urgent redo-sternotomy and replacement of the aortic arch was planned.

Redo median sternotomy was performed without incident. The axiallary and subclavian vessels were explored from cannulation however, the axiallary vessels were too small to sustain adequate antegrade bypass flows. Cannulation for cardiopulmonary bypass (CPB) was therefore achieved centrally with core cooling to 22^o^C. Dexamethasone administered at 27degrees C and ice packs to the head for cerebral protection. Antegrade cold blood cardioplegia was delivered into the aortic root at 22^o^C followed by initiation of circulatory arrest.

**Fig. 2:**
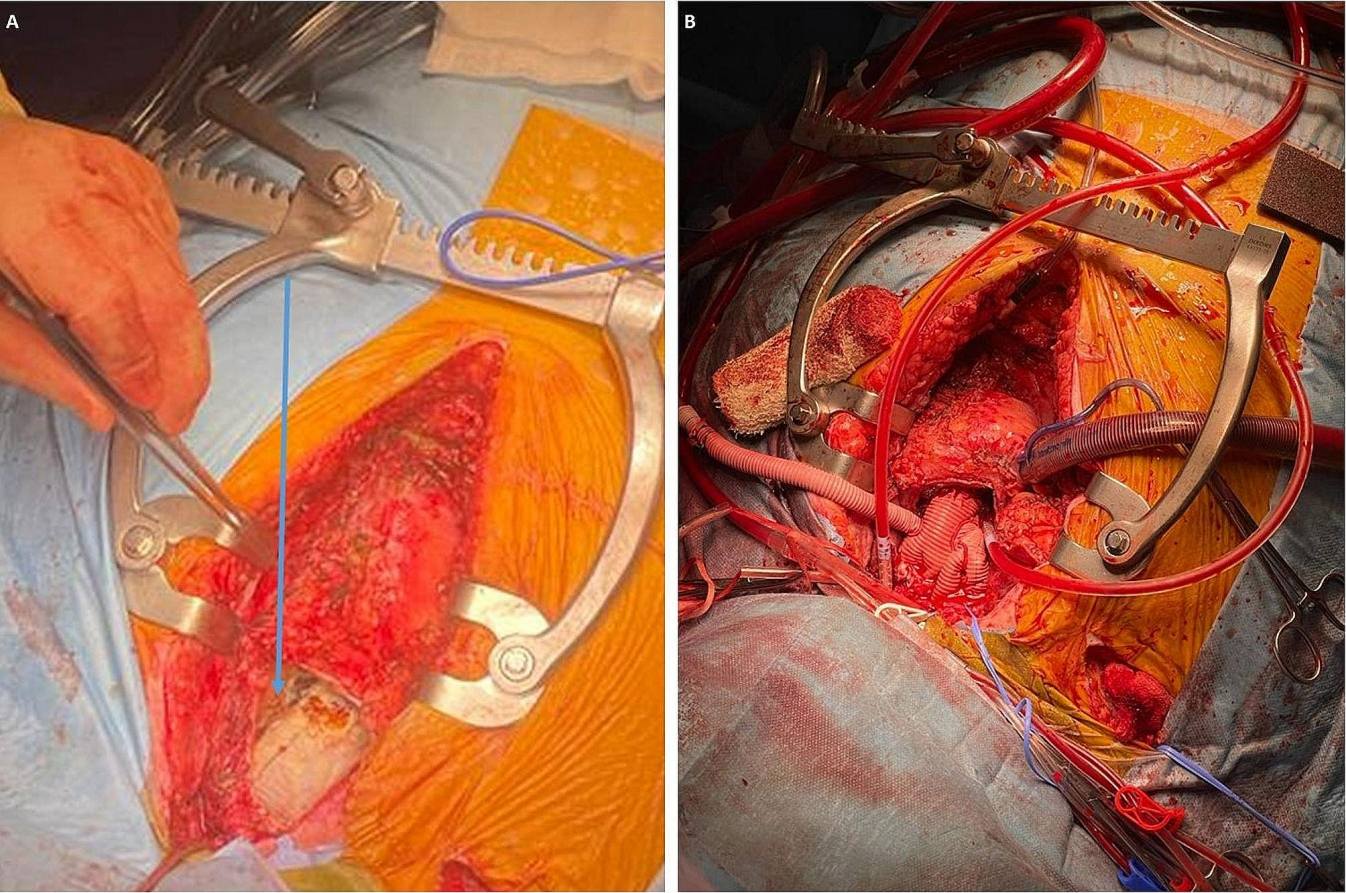
(**A**) Intraoperatively the suspected perigraft haematoma was actually a contained seroma, this was opened (blue arrow). (**B**) The repair involved a frozen elephant trunk with trifurcate graft. The original interposition graft was resected to leave a cuff just above the sino-tubular junction proximally. The Frozen Elephant Trunk device was deployed through the AMDS device and sutured in place. The arch vessels were de-branched and anastomosed to the trifurcate branches before a graft to graft anastomosis of the proximal and distal aortic grafts were completed

The arch vessels were dissected, and the previous ascending aortic graft was transected and trimmed back to the cuff of the AMDS stent distally. The suspected haematoma was found to be a seroma upon opening (Fig. 2A). The arch vessels were de-branched and ligated proximally. Self-expanding retrograde cardioplegia cannulae were inserted into the innominate and left common carotid arteries to deliver direct antegrade cerebral perfusion. The left subclavian artery was difficult to access, so it was occluded temporarily with a vascular clamp. A trifurcated frozen elephant trunk (FET) device (EVITA Open Neo) was deployed in through the AMDS and the covered stent expanded. The cuff was anastomosed to the previous Teflon cuff of the AMDS in a continuous fashion.

The arch vessels were anastomosed to the trifurcate branches (Fig. 2B). Distal body perfusion recommenced via arterial cannulation of the graft sidearm. The proximal end of the graft was trimmed. The previous ascending aortic graft was also trimmed to the region of the sinotubular junction. The proximal anastomosis was assessed for leaks with introduction of cardioplegia down the tube graft and found to be intact. Graft to graft anastomosis was then performed. The patient was weaned off CPB with inotropic and vasopressor support and the chest closed.

**Fig. 3:**
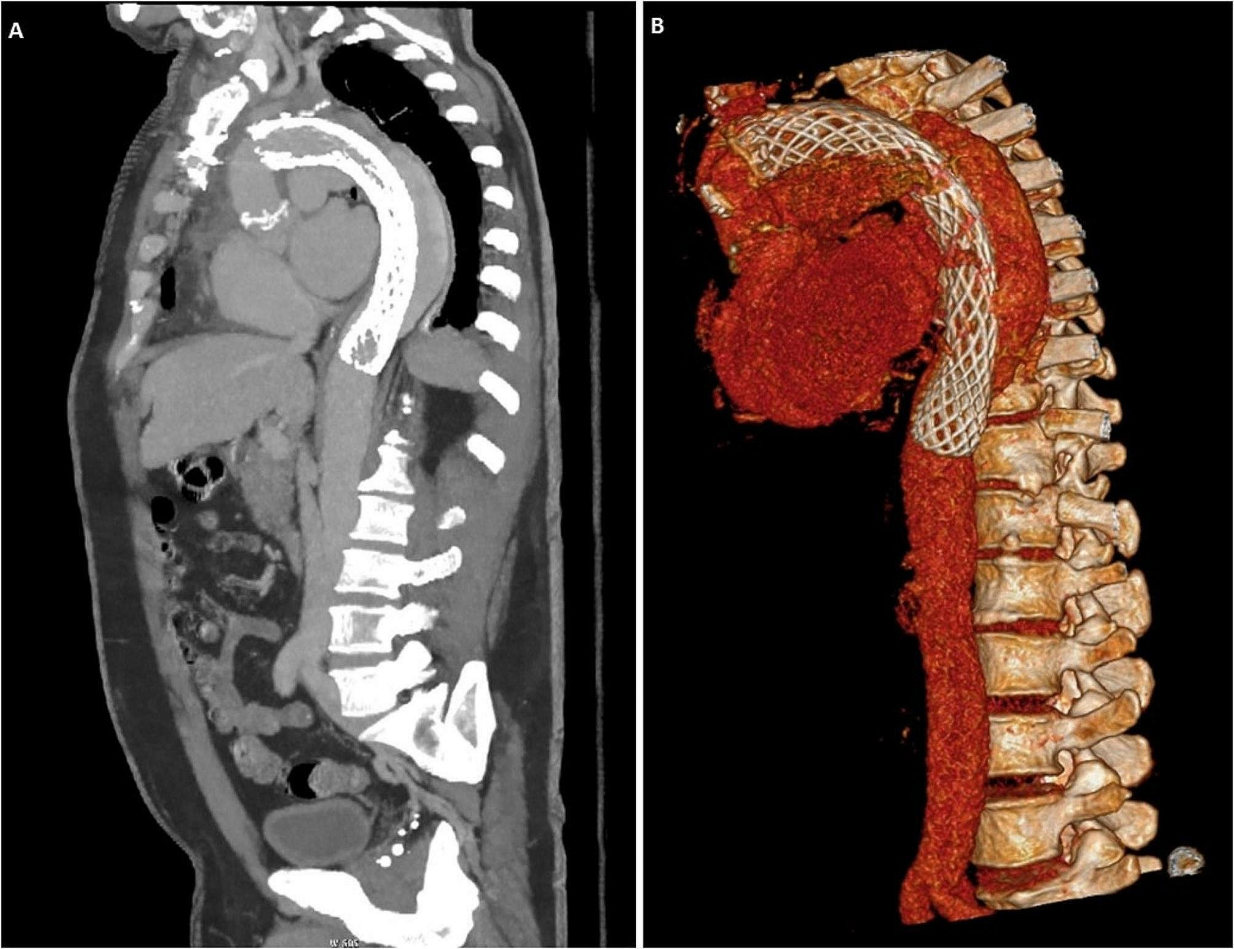
(**A**) CT aorta post repair showing resolution of the perigraft seroma with no contrast extravasation. (**B**) 3D reconstruction showing the FET within the AMDS

The post-operative CT aortogram showed a good repair with no false lumen opacification (Fig. 3). The patient was discharged and reviewed in the outpatient clinic without any issues and is for ongoing CT surveillance.

## Discussion

SAAD is the most common cardiac surgical emergency with a high burden of mortality and morbidity. Patients surviving emergency surgery are at an increased risk of ongoing downstream aortic degeneration. There has been a growing consensus that a more extended repair at the initial insult would limit this issue. However, concerns over acute operative outcomes may limit the extent of repair that is considered safe by the surgeon [4].

Distal anastomotic new entry (DANE) is considered to be an important cause of a persistent false lumen following repair of acute aortic dissection [5]. AMDS stent is a novel uncovered aortic arch hybrid graft implanted antegradely during hypothermic circulatory arrest to promote true lumen expansion and enhance aortic remodelling. Developed as an adjunct to standard surgical repair, it aims to improve malperfusion and promote remodelling of the aortic arch and distal dissected aorta at the time of initial surgery without complicating surgery [1].

The use of the AMDS is becoming more common in emergency surgery for acute aortic dissection, with a view to stabilising the true lumen and creating a landing zone for thoracic endovascular aortic repair (TEVAR) options. Use of the AMDS device requires careful pre-operative planning to avoid central AMDS collapse [6].

AMDS stent use is contraindicated in patients with genetic aortopathy and nickel allergies as per the manufacturer: They should be avoided when tears extend into the arch. These cases should be managed by resection of the arch tear or obliteration with a covered stent following debranching procedure due to the risk of an ongoing patent false lumen. Despite promising results, the unknown issues surrounding redo-surgery for the aortic arch as well as a yet to be proven long-term clinical safety and efficacy, total arch replacement remains the best evidence based practice in SAAD involving the arch [7].

Surgical options for total arch repair in cases where an AMDS device has been inserted are somewhat unknown. The device itself cannot be removed, which can cause a significant problem should surgical re-intervention be required, either due to a persistent false lumen or a leak. In this case, a covered endovascular stent was not an option due to the arch vessel patency and as such careful planning and multidisciplinary discussion helped to plan a surgical approach to debranch the arch and stabilise the false lumen. Re-operation with an AMDS in situ can present many technical challenges with the device also noted to distort the anatomy and create difficulty in accessing the arch vessels.

The AMDS device has ultimately shown promising results in studies so far in relation to aortic remodelling. As this case shows however, there are pitfalls to its use, including complicating the approach to further surgery involving the arch. Longer term data is required to clarify whether this arch management strategy improves late remodelling and reduces the need for further reintervention. The technical feasibility of reinterventions in the context of an in situ uncovered stent are also unknown however, we present one possible approach with a good outcome.

## Data Availability

Not applicable.

## Abbreviations

SAAD: Stanford-A aortic dissection
AMDS: Ascyrus Medical Dissection Stent
DARTS: Dissected Aorta Repair through Stent Implantation trial
CPB: Cardiopulmonary bypass
FET: Frozen elephant trunk
DANE: Distal anastomotic new entry
TEVAR: Thoracic endovascular aortic repair

## References

[1] Montagner M, Heck R, Kofler M, Buz S, Starck C, Sündermann S (2020). New Hybrid Prosthesis for Acute Type A aortic dissection. Surg Technol Int.

[2] Bozso SJ, Nagendran J, Chu MWA, Kiaii B, El-Hamamsy I, Ouzounian M (2021). Midterm outcomes of the dissected aorta repair through Stent Implantation Trial. Ann Thorac Surg.

[3] Bozso SJ, Nagendran J, MacArthur RGG, Chu MWA, Kiaii B, El-Hamamsy I (2019). Dissected Aorta Repair through Stent Implantation trial: Canadian results. J Thorac Cardiovasc Surg.

[4] Roselli EE, Vargo PR. Bare stenting of acute dissection: a gentle push forward. Eur J Cardiothorac Surg. 2023;63(3). 10.1093/ejcts/ezad039 36790074

[5] Tamura K, Chikazawa G, Hiraoka A, Totsugawa T, Sakaguchi T, Yoshitaka H (2017). The prognostic impact of distal anastomotic new entry after acute type I aortic dissection repair. Eur J Cardiothorac Surg.

[6] Luehr M, Gaisendrees C, Yilmaz AK, Winderl L, Schlachtenberger G, Van Linden A et al. Treatment of acute type a aortic dissection with the Ascyrus Medical Dissection Stent in a consecutive series of 57 cases. Eur J Cardiothorac Surg. 2023;63(3). 10.1093/ejcts/ezac581 36548434

[7] Al-Tawil M, Jubouri M, Tan SZ, Bailey DM, Williams IM, Mariscalco G (2023). Thoraflex Hybrid vs. AMDS: to replace the arch or to stent it in type A aortic dissection?. Asian Cardiovasc Thorac Ann.

